# Hope for the Future and Its Associated Factors Among Adolescents in Tanzania: A Cross-Sectional Study

**DOI:** 10.7759/cureus.68837

**Published:** 2024-09-06

**Authors:** Yasmine M Osman, Sanmei Chen, Naoki Hirose, Mariko Komada, Nicolaus Madeni, Frida Madeni, Yoko Shimpuku

**Affiliations:** 1 Nursing, Global Health Nursing, Graduate School of Biomedical and Health Sciences, Hiroshima, JPN; 2 Obstetrics and Gynaecology Nursing, Zagazig University, Zagazig, EGY; 3 Nursing, NPO Class for Everyone, Kanagawa, Tokyo, JPN; 4 Epidemiology and Public Health, Magunga Hospital, Korogwe, Tanga, TZA; 5 Research, The New Rural Children Foundation, Dar es Salaam, TZA

**Keywords:** adolescents, children and adolescence, future, hope, quantitative research, tanzania

## Abstract

Introduction

Hope is a universal, multidimensional psychological construct related to an individual’s expectation that they will achieve desirable outcomes by setting realistic objectives. The study aims to investigate the factors associated with hope among adolescents in Tanzania.

Methods

Baseline characteristics were collected from 2,058 adolescent girls and 1,770 adolescent boys at 41 primary and 13 secondary schools in Korogwe District, Tanzania*.* The level of hope was measured using the Herth Hope Index. The associated hope factors were analyzed using multiple linear regression models based on sex.

Results

The results of multiple regression analyses indicated that secondary school students demonstrated significantly higher levels of hope for the future than primary school students among both adolescent girls and boys [95% CI]: 2.34 [1.53 to 3.15], 1.69 (0.98 to 2.41). Both Christian adolescent girls and boys had significantly higher levels of hope than their Muslim counterparts. Adolescent girls who intended to enrol in secondary school or obtain employment exhibited significantly higher levels of hope than those who were uncertain about their post-school plans.

Conclusions

This study showed common and distinct factors associated with hope for the future ofadolescent girls and boys in Tanzania. This suggests that there are potential avenues for identifying target subpopulations of adolescent girls and boys and developing sex-specific interventions to enhance their hope for the future. Future research must focus on elucidating the methods for assessing and measuring hope in various populations in order to understand the essence of this concept based on strengths within particular cultural contexts.

## Introduction

Adolescence is a transitional phase between childhood and adulthood, characterized by increased capacity, responsibility, and opportunities [1]. Although adolescents can bring about significant change, many of them are not allowed to express their ideas or take action to achieve their desired objectives [2]. According to the WHO, adolescence is a critical period of life that comprises the transition from juvenile stages to adult stages, with ages ranging from 10 to 19 years. This period is critical for the development of a sense of direction, and age is merely one of many characteristics that define it [3]. These changes align with the escalating complexity of social and emotional experiences. However, the majority of issues that arise during the adolescent period are frequently overlooked, particularly in low-resource settings [4]. Consequently, numerous adolescents are susceptible to a variety of challenges in various domains, such as increasing academic demands, rearranging relationships with parents and peers, and developing one’s identity [5].

Tanzania is a developing country, with 36% of its population residing below the national poverty line. Tanzania has experienced remarkable economic growth over the past decade; however, poverty has not decreased as much as the population has grown, increasing the absolute number of impoverished individuals [6]. In 2018, approximately 14 million individuals were living below the national poverty line of TZS 49,320 per adult equivalent per month, while approximately 26 million individuals (approximately 49 percent of the population) were living below the international poverty line of $1.90 per person per day [7,8].

In Tanzania, adolescents account for 23.6% of the total population [9]. In addition, they are exposed to a variety of risks as they mature, including early marriage, sexually transmitted infections, HIV, and violence. These risks are frequently linked to poverty at the household and community levels. In this context, the promotion of adolescent health in Tanzania is becoming increasingly urgent [10]. Health promotion efforts are increasingly focused on comprehending the interdependent changes during adolescence. These changes encompass physiological and cognitive developments, shifts in social environments and interpersonal relationships, as well as behavioral and psychological adjustments [11]. Teenagers consequently develop a greater capacity for setting clear objectives, making advance plans, and carrying out the necessary actions to accomplish these goals. These developmental advances advancements have the potential to establish adolescence as the optimal period for the cultivation of numerous psychological strengths, such as hope [12].

In the past few years, hope has become one of these constructs that has gained popularity. This construct is defined as a multidimensional positive motivational construct that includes future-oriented beliefs about one’s ability to achieve goals and overcome obstacles [13-16]. Over the past twenty years, In order to gain a comprehensive understanding of and focus on adolescent life over the past twenty years, researchers have endeavored to conceptualize and measure the level of hope in the context of mythology, religion, philosophy, education, and science [17]. Tanzania’s socioeconomic landscape, which is characterized by regional variations, economic disparities, and cultural diversity, has a significant impact on the experiences and aspirations of adolescents [18,19].

According to Snyder’s theory, people engage in cognitive and motivational processes that translate into mental action sequences to accomplish their goals [20]. Snyder’s theory posits that individuals engage in cognitive and motivational processes that result in mental action sequences to achieve their objectives [21]. Individuals with high expectations are confident in their established path and are adaptable in finding alternative or different ways to achieve their objectives [22]. Individuals’ perception of their capacity to produce these components is the cornerstone of hope theory. Individuals with high hopes are confident in their ability to start and maintain progress toward their objectives (agency thinking) and to establish feasible pathways to achieve these objectives (pathways thinking). Therefore, experts consider optimism essential for adolescents’ development, particularly in the context of identity formation and future planning [23,24].

Hopelessness in adolescence is predictive of a broad range of risk behaviors, including violent and aggressive behavior, substance abuse, sexual behavior, and accidental injury [25,26]. In addition to fewer externalizing behaviors, higher hope for the future is associated with more favorable outcomes, such as self-esteem, life satisfaction, and academic success [27]. Researchers have emphasized the significance of emphasizing the positive outcomes of hope, mediating the effects of threat and challenge on orientation toward the future [28]. The focus has been on the future and the positive effects of hope, thereby mediating the influence of threat and challenge on one’s perspective on the future [29]. Conversely, depression and suicidal ideation are linked to a lack of hope or hopelessness [30,31].

Few studies have been conducted in Tanzania. In this study, a baseline survey was conducted to contextualize and enhance our understanding that adolescence is a critical developmental phase that presents unique challenges and opportunities. Prior studies did not examine adolescents’ hope for the future and its associated factors. The current study provides valuable insights since it is the first study conducted among adolescents in Tanzania and early and late adolescent students. Additionally, it is crucial to distinguish between boys and girls in Tanzania in order to comprehensively explore their hopes for the future. To gain an understanding of the factors that influence hope for the future, this study aimed to investigate hope for the future and its associated factors among adolescent girls and boys in Tanzania.

Study objectives

The current study aims to investigate hope for the future and its associated factors among adolescent girls and boys in Tanzania. Additionally, the study aimed to examine the factors that contribute to gender differences in hope for the future among adolescent girls and boys in Tanzania.

## Materials and methods

Conceptual framework

**Figure 1:**
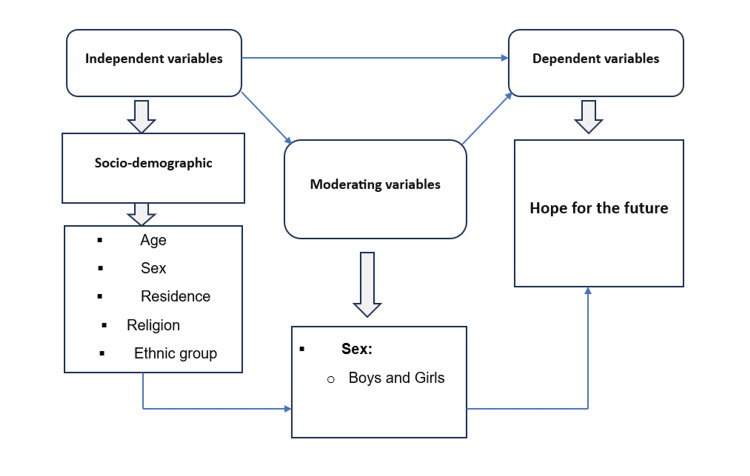
Conceptual framework of the study

A wide variety of factors are associated with hope for the future, as indicated by previous literature. Nevertheless, there is a lack of a conceptual framework that unites these numerous factors and elucidates their combined effect on adolescents. Figure 1 presents a conceptual framework that aims to comprehend the complex interplay between sociodemographic factors such as age, sex, religion, ethnic group, and hopes while considering moderating variables (such as sex). This framework can offer valuable insights into the nuanced nature of individual perspectives on the future. In addition, it offers a more profound comprehension of the dynamics of hope in various demographic contexts.

Study design and participants

**Figure 2:**
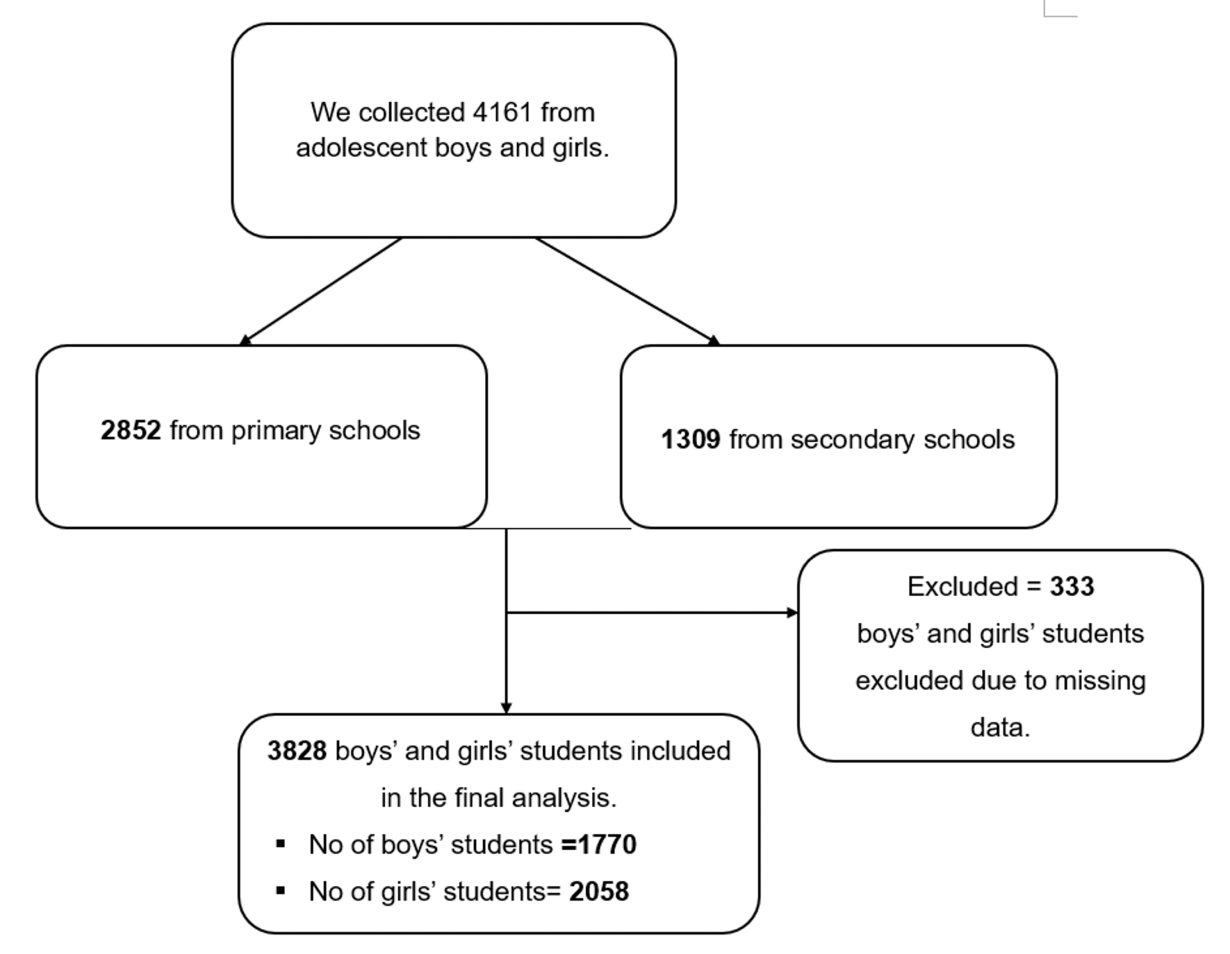
Flow chart of the study sample

This cross-sectional study used data from a survey conducted between April 1, 2017, and June 30, 2017, among students in primary and secondary schools in the Korogwe District in Tanzania [32]. The study participants consisted of male and female students from 41 primary schools and 13 secondary schools located in the rural area of Korogwe District, Tanzania, in the northeastern part of the country. We administered the instruments in classroom settings. The selected school principals were provided with information regarding the study during information sessions. The study’s objectives and the survey’s content were elucidated to the teachers, who subsequently conveyed this information to the students. Comprehensive guidance on how to react to each instrument and details about the study were given during the administration of the instruments. The researchers or educators at each school administered a survey to all intended students, encompassing both male and female individuals. Each student was individually responsible for completing the questionnaire. The study sample’s assembly is depicted in Figure 2). We collected data from 2,852 students attending primary schools and 1,309 students attending secondary schools. Consequently, the final sample comprised a total of 2,058 adolescent girls and 1,770 adolescent boys. All participants provided their consent and were treated in compliance with the ethical guidelines specified in the Declaration of Helsinki and its subsequent revisions. Our study was part of the Japan International Cooperation Agency (JICA) Partnership Program provided by NPO Class for Everyone, Japan. The New Rural Children Foundation, a local collaborator, assisted with school arrangements and data collection. This study received ethical approval from the National Institute of Medical Research in Tanzania (NIMR/HQ/R.8a/Vol.IX988). Following that, the data underwent a cleaning and screening process to ensure the data input’s accuracy and prepare it for analysis.

Measurements of the hope for the future

The Herth Hope Index (HHI) was utilized to measure the level of hope for the future (see Appendices). This index was developed by Herth [33], and it measures various dimensions of hope levels. The scale has been extensively utilized in both clinical and research settings. It consists of 12 items, using a 4-point Likert scale ranging from 1 (strongly disagree) to 4 (strongly agree), with two reverse-coded items. Since the original scale was English, we obtained permission from Herth to translate it into Kiswahili. Tanzanian researchers proficient in both English and Kiswahili carried out a back-translation process, and the final version was verified by the last author, who is fluent in both languages. The scale is measured by a single global score that ranges from 12 to 48 points, with a higher score denoting higher levels of hope for the future. We calculated the total score by adding the scores of each item. There is a detailed description of the items comprising the hope score in the Results section. HHI demonstrated a high degree of divergent validity, as well as robust construct validity. As part of the revised article, we have added a detailed section describing the scale's reliability. To determine the scale's internal consistency, Cronbach's alpha was used. The total number of the scale was 12 items. The Cronbach's Alpha reliability coefficient of the full scale was 0.783.

Measurements of correlates of hope for the future

The questionnaire gathered data on the baseline characteristics of adolescents, including variables such as age, sex (males or female), sibling status (yes or no), religion (Christian, Muslim, or other), ethnic group (Msambaa, Mchaga, Mzaramo, or other), and the occurrence of menstruation or nocturnal emission (yes or no). Have you engaged in sexual intercourse previously (yes or no)? Do you anticipate getting married in the future (yes or no)? Furthermore, we categorized each student from the school data based on their educational level (primary or secondary).

Statistical analysis

Participants’ characteristics were described as mean and standard deviation for continuous variables and percentage for categorical variables according to sex. Model assumptions for linearity, normality, and homoscedasticity were tested. Given that these conditions were adequately fulfilled, we performed multiple linear regression analyses to investigate potential factors associated with hope for the future. In these multiple regression analyses, we used the variance inflationary factor (VIF) test to eliminate the possibility of multicollinearity among the independent variables. Multiple linear regression analyses were conducted based on sex to account for potential variations in the association between independent variables and hope for the future. All statistical analyses were conducted using the Statistical Package for the Social Sciences (SPSS) (version 26; IBM Corp., Armonk, USA). A two-tailed p-value <0.05 was considered statistically significant.

## Results

A total of 2,058 adolescent females and 1,770 males from 41 primary schools and 13 secondary schools were included in the final sample. Table 1 shows the sociodemographic characteristics of the study participants differentiated by sex. The mean (SD) age of students was 13.10 ±1.699 years for adolescent girls and 13.55 ±1.679 years for adolescent boys. 67.1% of whom were from primary schools and 32.9% of whom were from secondary schools. In terms of sex, 46.2% were males and 53.8% were females. Regarding the ethnic group, 51.1% of them were from the Msambaa group. Moreover, 77.2% of the adolescents answered that they had hope when queried about their prospects for marriage in the future. The mean (SD) score of hope for the future was 39 ±4.99 for adolescent girls and 39 ±4.465 for adolescent boys and the overall mean of the hope score was 39 ±4.75 with no significant differences.

**Table 1:** Characteristics of participants according to sex The mean ± standard deviation SD for continuous variables and as numbers N with percentages % for categorical variables. Independent t-tests for continuous variables and chi-square tests for categorical variables. The level of statistical significance was set at p<0.05.

Variable	Model	Male (N=1770)		Female (N=2058)		Total (N=3828)	
		N (%)		N (%)		N (%)	
Age In Years	Mean± SD	13.55 ±1.679		13.10 ±1.699		13.31 ±1.704	
	Primary	1213	68.5	1356	65.9	2569	67.1
	Secondary	557	31.5	702	34.1	1259	32.9
Have Brother/Sister, N (%)							
	Yes	1528	86.3	1849	88.2	3377	88.2
	No	242	13.7	209	10.2	451	11.8
Religion, N (%)							
	Christian	707	39.9	849	41.3	1556	40.6
	Muslim	1054	59.5	1196	58.1	2250	58.8
	Others	9	0.5	13	0.6	22	0.6
Plan after Finishing School (Career), N (%)	I don’t know	40	2.3	48	2.3	88	2.3
	I will find a job	636	35.9	693	33.7	1329	34.7
	I will go to secondary school	1094	61.8	1317	64	2411	63
Hope for Marriage	Yes	1440	81.4	1514	73.6	2954	77.2
	No	330	18.6	544	26.4	874	22.8
Race, N (%)	Msambaa	962	54.4	96	4.7	1958	51.1
	Mchaga	82	4.6	256	12.4	178	4.6
	Mpare	198	11.2	39	1.9	454	11.9
	Mzaramo	37	2.1	671	32.6	76	2.0
	other	491	27.7	96	4.7	1162	30.4
Total Hope Score	Mean± SD	39 ±4.465		39 ±4.99		39 ±4.75	

Table 2 shows the results of multiple linear regression analyses stratified by sex. In comparison primary school students exhibited significantly higher levels of hope for the future among both adolescent girls and boys compared to their counterparts in the secondary school students [95% confidence interval]:1.69 [0.98 to 2.41], p < 0.001, respectively). Christian adolescent girls and boys exhibited significantly greater levels of optimism for the future compared to their Muslim counterparts [95% confidence interval]: 1.09 (0.66 to 1.51), p < 0.001; 1.22 [0.81 to 1.64], p < 0.001, respectively). Adolescent girls who had a plan for their future after finishing school, specifically those who planned to attend secondary school or find a job, had significantly higher levels of hope for the future compared to those who did not have a plan [95% confidence interval]: 2.58 [1.19 to 3.97], p < 0.001; 1.93 (.50 to 3.37), p = 0.008, respectively). Among adolescent boys, those who identified as Mchaga (versus Msambaa) and those who expressed a lack of desire to get married (versus yes) exhibited significantly lower levels of hope[95% CI]: -1.51 [-2.49 to -0.52], p = 0.003; -0.74 (-1.27 to -0.22), p = 0.006, respectively).

**Table 2:** Multiple linear regression analyses stratified by sex. The unstandardized coefficients, Standardized coefficients, p-values, and 95% confidence intervals 95% CI. Ref indicates the reference category. The analyses were conducted using multiple linear regression.

Sex	Variable	Model	Un-std. Coefficients		Std. Coefficients	95% CI		P value
			B	Std. error				
Male	School	Primary	1.695	.363	.176	.984	2.407	.000
		secondary	Ref	Ref	Ref	Ref	Ref	
	Age	(one unit increment)	.148	.086	.056	-.021	.317	.085
	Having brothers or sisters	Yes	-.312	.300	-.024	-.900	.277	.299
		No	Ref	Ref	Ref	Ref	Ref	
	Sexual intercourse experience	Yes	1.131	1.166	.022	-1.156	3.417	.332
		No	Ref	Ref	Ref	Ref	Ref	
	Hope for marriage	Yes	-.743	.268	-.065	-1.269	-.217	.006
		No	Ref	Ref	Ref	Ref	Ref	
	Religion:	Muslim	1.222	.211	.134	.807	1.636	.000
		Other	.661	1.456	.011	-2.195	3.518	.650
		Christian	Ref	Ref	Ref	Ref	Ref	
	After finishing school	I will find a job	-.265	.280	-.029	-.814	.283	.343
		I don’t know	-1.301	.698	-.043	-2.669	.068	.062
		I will go to secondary school	Ref	Ref	Ref	Ref	Ref	
	Race	Mchaga	-1.506	.500	-.071	-2.487	-.524	.003
		Mpara	-.098	.338	-.007	-.761	.564	.772
		Mzaramo	-.525	.724	-.017	-1.945	.896	.469
		Others	.409	.240	.041	-.062	.880	.089
		Msanbaa	Ref	Ref	Ref	Ref	Ref	
Female	Variable	Model	Un-std. Coefficients		Std. Coefficients	95% CI		P value
			B	Std. error				
	School	Primary	2.343	.414	.222	1.531	3.154	.000
		secondary	Ref	Ref	Ref	Ref	Ref	
	Age	(one unit increment)	.186	.100	.063	-.010	.381	.063
	Have brothers or sisters	Yes	.003	.353	.000	-.689	.695	.994
		No	Ref	Ref	Ref	Ref	Ref	
	Sexual intercourse experience	Yes	-.149	2.156	-.001	-4.377	4.079	.945
		No	Ref	Ref	Ref	Ref	Ref	
	Hope for marriage	Yes	-.387	.247	-.034	-.871	.098	.118
		No	Ref	Ref	Ref	Ref	Ref	
	Religion	Muslim	-.084	1.343	-.001	-2.718	2.550	.950
		Other	1.087	.217	.107	.661	1.513	.000
		Christian	Ref	Ref	Ref	Ref	Ref	
	After finishing school	I will find a job	-.646	.297	-.061	-1.228	-.065	.029
		I don’t know	-2.580	.709	-.078	-3.970	-1.191	.000
		I will go to secondary school	Ref	Ref	Ref	Ref	Ref	
	Race	Mchaga	-.014	.516	-.001	-1.026	.999	.979
		Mpara	.336	.337	.022	-.326	.997	.320
		Mzaramo	.434	.785	.012	-1.105	1.973	.580
		Others	.424	.244	.040	-.055	.902	.083
		Msanbaa	Ref	Ref	Ref	Ref	Ref	

## Discussion

The concept of hope for the future is essential for achieving one’s life goals and success [34]. The main objective of our study is to investigate the level of hope for the future and associated factors among male and female adolescent students in Tanzania. This study employed multiple regression analyses that were stratified by sex. The data collected and analyzed using the Herth Index to measure hope revealed that this study found both shared and unique factors influencing the hope for the future of adolescent girls and boys in Tanzania. This study has yielded statistically significant findings regarding the level of hope for the future among adolescent girls and boys in Tanzania. Therefore, the contribution of this study helps understand the hope for the future among adolescent student and the associated factors that affect their level of hope.

In this study, we discovered that the level of hope for the future was significantly higher among adolescent primary school students than their counterparts in secondary school for both adolescent girls and boys. The current findings align with a prior investigation conducted by Botor [35], which demonstrated substantial levels of hope among male and female adolescents in secondary school. Another study conducted by Fraser et al. [36] found a significant decrease in hope levels among primary school students before the transition to high school.

The present results are also consistent with previous studies [37-39], which indicated that the academic stage in school plays a significant role in shaping hope for the future. Given the influential role of parental interactions in the development of young adolescents transitioning from late childhood, it is unsurprising that they continue to have high levels of hope based on their interactions and support from their families [40]. During the early stages of adolescence, individuals frequently experience a strong desire to conform to societal norms and are highly sensitive to embarrassment [41]. The variation in levels of hope during childhood and adolescence in school is often attributed to the individual’s exposure to stressful life events throughout their lifetime [42]. 

Additionally, Synder [43] found that hope declines from late childhood to adolescence in Portuguese children. The decline in hope for the future among primary school students could be partly explained by simultaneously experiencing rapid cognitive, physical, emotional, and social changes [44,45]. This provides insight into the disposition of the participants, and the literature shows that late adolescents in secondary school have a unique set of concerns and characteristics that are different from early adolescents in primary school [46,47].

Our findings from this study indicate that the students’ hope toward the future was affected by their religiosity as we found that both Christian adolescent boys and girls had higher levels of hope for the future compared to Muslim adolescent students. Our finding is consistent with a previous study [48-50]. In contrast, the study conducted by Parkins [51] to investigate hope among Tanzanian adolescents in a school environment found no notable variations in hope scores among participants with varying religious beliefs. According to Ciarrochi and Heaven’s study, religious values appear to have an impact on adolescents’ well-being. Another study conducted by Tiliouine [52,53] demonstrated that religious belief and religious altruism play a significant role in offering hope and meaning in the lives of Muslims. Furthermore, religious values may affect adolescents’ hope for the future and might have an important role in promoting those characteristics, thereby enhancing hope [54].

Our findings indicate that adolescent girls who had intentions to pursue secondary education or seek employment displayed significantly higher levels of hope for the future compared to those who did not have a specific plan, but we failed to observe this association among adolescent boys. Our results align with the findings of a study conducted on a group of teenagers from North America [55], which indicated that girls perceive support from their parents and family in various ways. Furthermore, adolescents are generally more receptive to social support and relationships [56].

Studies on the impact of racial identity on hope have yielded contradictory results. Our study revealed a significant correlation between being of the Mchaga race and lower scores for hope in the future among adolescent boys but not in adolescent girls. A previous study [57] found that during middle and high school, African Americans reported the highest levels of hope, followed by Caucasians in second place, while Hispanic middle school students reported the lowest levels of hope. Conversely, Change [58] found no significant differences between groups. This finding contradicts the results of the previous study conducted on US samples. In Tanzania, traditional family structure may reveal different childrearing practices depending on children’s gender [59]. Boys often develop autonomous life views and styles, whereas girls tend to be more controlled by their significant others than boys [60].

Furthermore, male students who expressed hope for marriage had significantly lower levels of hope for the future. However, this association was not detected among female students. In this area of Tanzania, the benefits of marriage may may render early marriage appealing to certain young women, as it serves as a way to gain respect and status within one’s community [61]. A qualitative study conducted in rural Tanzania reported that marriage is viewed as a way of becoming an adult, gaining status within the community, and providing financial support and honor to their families. Marriage is also occasionally viewed as a means of alleviating the risks of sexually transmitted diseases associated with adolescence [62]. Furthermore, according to the tradition of child marriage in Tanzania, girls are routinely portrayed as forced into marriage for the economic gain of their parents [63]. This finding may elucidate why adolescent males need to demonstrate greater responsibility and self-reliance to successfully complete their education, thereby gaining economic autonomy and social security.

However, some studies focus on the Hope scale rather than participants’ gender. Some studies focus on gender and have shown significant and non-significant gender differences in adolescents. Previous literature [64,65] found that females and males demonstrated different hope levels throughout adolescence, with males reporting higher levels of hope than their female counterparts. Conversely, Hartanto et al. [66] found that adolescent females expressed higher hope than males. Furthermore, another study has shown non-significant gender differences among adolescents [67].

Strengths and limitations of this study

The study has some strengths. This study is the first in Tanzania, as there is a scarcity of research on adolescent hope for the future in the country. Second, the sample size is large. In addition, the study provides valuable insight into the significant sociodemographic differences in adolescent hope and essential and unique information regarding hope for the future and its related factors.

This study had limitations that need to be considered when interpreting the findings. First, the study results cannot be generalized to other adolescent populations because the study was conducted within one district of Tanzania. Future research should attempt to replicate our findings among diverse adolescent populations. Second, because the study design was cross‐sectional, we were unable to investigate the potential relationship between the associated factors and changes in hope for the future over time, and our findings do not imply causality. In order to gain a deeper understanding of the variability of hope patterns within individuals over time, it is recommended that future research investigates these relationships longitudinally. Furthermore, we did not have sufficient data on additional potential factors associated with hope for the future among adolescent girls, which could potentially impact the level of hope among adolescent students. These factors include the sociodemographic data of parents, such as their income and education levels. Therefore, future research is needed to examine these factors.

## Conclusions

This study identified both common and distinct factors associated with hope for the future among adolescent girls and boys in Tanzania. Our findings suggest potential approaches for identifying target subpopulations of adolescent girls and boys with low levels of hope for the future. This can help develop interventions tailored to their needs and aimed at improving their hope for the future. The potential for hope can be enhanced if adolescents are enrolled in higher education and can be influenced by sex and future goals. Future research should examine the specific variations in goal setting, particularly among adolescents in primary schools. Intervention programs should consider investigating how young individuals can utilize their thoughts about family, peers, and spiritual resources as motivators to achieve their goals. In order to enhance the levels of hope among female students in Tanzania, school counsellors need to consider both personal emphasis and gender role attributions.

## Appendices

**Table 3:** Herth Hope Index (HHI) Scale

Items	Strongly disagree	Disagree	Agree	Strongly agree
I have a positive outlook toward life.	1	2	3	4
I have short and-or long-range goals.	1	2	3	4
I feel all alone.（reverse item）	1	2	3	4
I can see possibilities in the midst of difficulties.	1	2	3	4
I have a faith that gives me comfort.	1	2	3	4
I feel scared about my future. （reverse item）	1	2	3	4
I can recall happy/ joyful times.	1	2	3	4
I have deep inner strength.	1	2	3	4
I can give and receive caring/ love.	1	2	3	4
I have a sense of direction.	1	2	3	4
I believe that each day has potential.	1	2	3	4
I feel my life has value and worth.	1	2	3	4
